# Core Moral Concepts and the Sense of Fairness in Human Infants

**DOI:** 10.1007/s12110-025-09490-0

**Published:** 2025-04-02

**Authors:** Luca Surian, Eugenio Parise, Alessandra Geraci

**Affiliations:** 1 Department of Psychology and Cognitive Science, University of Trento, Trento, Italy; 2 CIMeC - Center for Mind/Brain Sciences, University of Trento, Trento, Italy; 3 Department of Educational Sciences, University of Catania, Catania, Italy

**Keywords:** Fairness, Core Knowledge, Moral Judgment, Justice, Infancy, Cognitive Development

## Abstract

We review recent experimental studies relevant to assess the proposal that human infants possess a sense of fairness that relies on sociomoral knowledge. We propose that this knowledge may include a core concept of justice with four foundational aspects: impartiality, agency, obligatoriness and conflicting claims. Infants’ and toddlers’ looking times, manual preferences and spontaneous actions provide some evidence for the first three features. Very early-emerging sociomoral evaluations and expectations about resource distributions show that infants process morally relevant information about distributors and recipients, suggesting that they are sensitive to the agency and impartiality constraints. Early evaluations appear to be linked to third-party expressions of praise or admonishment and to the deliverance of rewards and punishment, providing initial support for the obligatoriness constraint. More work is needed to investigate the sensitivity to conflicting claims, to assess the universality of early emerging evaluation skills and to show how core concepts relate to the development of explicit judgments and beliefs about duties and rights.

On a lucky day, one of us was taking his daughter to her kindergarten. Suddenly she shouted: “It is not fair!” (original: “*Non è giusto!*”, in her native Italian). “The teacher has favorites in class!”. The temptation to ask her a test question on ‘authority independence’’ (Turiel, 1983) was irresistible: “Well, Sofia, the teacher is the boss in class, isn’t she? So, why is it not right for her to have favorites, even if she likes to do so?” She pondered for a moment and then replied, “Because the other children will feel bad!”. While still unable to tie her shoes by herself, she could already articulate a protest against authorities’ unfairness!

How do children get to this so fast? Do they acquire an understanding of fairness at around 3 years (Engelmann & Tomasello, 2019; Vaish & Tomasello, 2023) through collaborative interactions with other children and adults (e.g., Kohlberg, 1971; Piaget, 1932/1965; Smetana, 1983), cultural inculcation (Prinz, 2006), and other domain-general learning mechanisms (e.g., Dahl, 2019; Mammen & Paulus, 2023)? Or, rather, is the ontogenetic process bootstrapped by innate sociomoral skeletal knowledge, evolved tacit principles and domain-specific developmental mechanisms (e.g., Baillargeon et al., 2015; Hamlin, 2013a; Premack & Premack, 1997; Sheskin et al., 2014; Sperber & Baumard, 2012; Wynn & Bloom, 2014)? The main goal of this article is to review recent evidence that bears on these questions and allows an initial assessment of the claim that humans possess an early emerging sense of fairness that relies on a core concept of justice. This knowledge would play a key role in the development of explicit reasoning in the domain of distributive fairness (Deutsch, 1975), as well as in other domains of justice such as retributive (dealing with the delivery of deserved punishments and rewards; Dunlea & Heiphetz, 2020) and procedural fairness (dealing with how to take decisions in order to treat people fairly; Shaw & Olson, 2014).

## Core Moral Concepts

Early emerging, possibly innate, core concepts and abstract principles help infants’ to spontaneously acquire, quickly and spontaneously, sophisticated competencies about numbers, objects, places, agents, social beings and mental states (Baillargeon et al., 2016; Carey, 2009; Leslie, 1994; Spelke, 2022). Some authors have broadened this core knowledge view to account for the acquisition of a moral competence (e.g., Baillargeon et al., 2015; Dwyer et al., 2010; Hamlin et al., 2007; Ting et al., 2020), positing innate principles of harm avoidance, fairness, group loyalty and authority that constrain infants’ reasoning and evaluation of agents and actions (Bian et al., 2018; Geraci & Surian, 2011; Haidt & Joseph, 2007; Margoni et al., 2018; Pellizzoni et al., 2010; Woo & Hamlin, 2023). Others have rejected this proposal, emphasizing the effects of early experiences and the role of learning mechanisms (Sommerville, 2023), or the viability of alternative explanations based on other core knowledge systems (Spelke, 2022, 2023; for replies see Hamlin, 2023; Revencu & Csibra, 2023). The implausibility of an evolutionary and modular view has also been pointed out. Carey (2009; p. 8) for example, states: “evolution did not provide us with an input analyzer that identifies electrons, *justice*, or 3,462,179” (emphasis added).

Here we will review recent developmental findings that reveal the early emergence of what people, possibly anywhere in the world, consider a central component of morality: the ability to tell just from unjust actions (Mikhail, 2022). We will cite most of the relevant works on infants (individuals aged 1 to 18 months) and toddlers (19-to-30 months-olds), as well as few studies on older children and adults. Due to space limitations, we will not review the evidence on non-human animals (e.g., Brosnan & de Waal, 2003), and will only briefly refer to relevant cross-cultural works on older children and adults belonging to small-scale societies and cultures classified as non-WEIRD (i.e., white, educated, industrialized, rich, and democratic).

People typically reason and talk about fairness and justice in their attempts to solve conflictual situations and to evaluate many different things including laws, institutions, procedures, traditions, societies, actions and persons. Ancient Greek, Roman and medieval philosophers saw justice (‘*justitia’*) as one of the most important virtues and, in line with this tradition, some psychologists consider it now a major strength of character (Peterson & Seligman, 2004). For Rawls (1958; 1971), justice is the first virtue of social institutions and fairness is its essential feature. Shifting the attention from virtues to actions, the ability to reason explicitly and autonomously about justice has been the main focus of classic theories of moral development (Damon, 1977; Kohlberg, 1971; Piaget, 1932/1965). Fairness and justice are terms used, sometimes interchangeably, with many different meanings. To avoid some confusions, it may be useful to start by saying that here we will focus on fairness and justice as properties attributed to actions and people, mainly in contexts involving distributions of resources (distributive justice), but also in contexts involving procedures, rewards and punishments (procedural and retributive justice).

Following Aristotle, many theories have emphasized that the common-sense conception of justice implies an idea of equality and fairness (Sen, 2009; Crisp, 2014). We may find a very good candidate for a definition of this concept in a sixth-century AD codification of Roman Law, the *Institutes of Justinian* (Miller, 2021) where justice is defined as ‘the constant and perpetual will to render to each his due’. In this definition, we can identify four important features: 1) *Impartiality (fairness)*: justice is connected with the impartial, application of a rule or principle, it is understood as the opposite of arbitrariness; 2) *Agency*: justice requires that there is an agent that alters the states of other agents, evaluations of fairness are therefore linked to the understanding of agents and the representation of their rights and duties (a natural disaster can be judged as bad, not as unfair); 3) *Obligatoriness*: justice involves an obligation to each person, it is demanded, not begged; and 4) *Conflicting claims and interpersonal conflicts*: people reason about justice typically in contexts that involve conflicts and conflicting claims about how to divide and share utilities, such as resources, freedom or opportunities. Does the empirical evidence provided by recent infant studies meet a criterion based on these features?

## Impartiality

Numerous recent studies investigated infants’ sense of fairness by presenting them with scenarios involving actions that violate or conform to *distributive justice* principles based on equality (egalitarian rules), or merit and deservingness (equity rules). According to Engelmann and Tomasello (2019), unfair actions may acquire their high negative value not out of a concern for the material benefits, but because they convey that someone is not getting the deserved respect (in the sense of what Darwall (1977; p. 38) called ‘recognition respect’: “giving appropriate consideration or recognition to some feature of its object in deliberating about what to do”). Infants were shown events involving simple resource distributions (for example, a cartoon animal distributes colored disks or strawberries to two other agents) and their looking times, helping preferences or manual choices have been recorded. The recipients were cartoon animals (e.g., Geraci & Surian, 2011); human adults (e.g., Schmidt & Sommerville, 2011), puppets (e.g., Bian et al., 2018; Sloane et al., 2012) or self-moving geometrical shapes with eyes (e.g., Meristo & Surian, 2013). The Violation of Expectation (VoE) paradigm provided the rationale for most of these experiments: longer looks at events that violate an expectation or a principle, compared to events that conform to it, suggest that infants generated expectations or inferences based on such a principle (for a conceptual overview see Margoni et al., 2024). In order to interpret the observed patterns of looking times as resulting from an expectation violation and to rule out alternative, more conservative explanations based on affiliative biases or low-level factors such as familiarity and perceptual symmetry, VoE studies typically include various control conditions (for a cogent discussion, see Hamlin, 2023; see also Stahl & Feigenson, 2019), or rely on convergent neuroimaging evidence (e.g., Forgács et al., 2020; Hudac & Sommerville, 2020; Southgate & Vernetti, 2014).

Infants display a variety of reactions to events involving resource distributions. All things being equal, infants and toddlers look longer at unequal than at equal distributions, suggesting that they, like preschoolers (Fehr et al., 2008; Elenbaas, 2019), expect agents to be impartial and follow an egalitarian rule to distribute windfall resources (e.g., Buyukozer Dawkins et al., 2019; Geraci & Surian, 2023a; Meristo et al., 2016; Schmidt & Sommerville, 2011; Sloane et al., 2012). Moreover, their manual choices reveal a reliable preference for fair over unfair agents (e.g., Geraci & Surian, 2011; Lucca et al., 2018), suggesting that they tag fair agents more positively than unfair ones (see also Burns & Sommerville, 2014). Consistent with this preference, toddlers also selectively help (Surian & Franchin, 2017a), share their own goods (Nava et al., 2019), accept a toy from (Burns & Sommerville, 2014) and reward fair agents (Ziv et al., 2021) rather than unfair ones.

Some studies have included control conditions in which resources are revealed rather than distributed (e.g., Meristo et al., 2016; Sloane et al., 2012) and they have consistently found that infants do not look longer at unequal allocations, they look longer only at unequal distributions, suggesting that they are not expecting that similar individuals have similar amounts of resources, rather they generate expectations about the distributive actions.

Toddlers can also rely on an equity principle: their looking times reveal that they expect resources to be distributed according to recipients' relative merit and deservingness (Sloane et al., 2012; Surian & Franchin, 2017b; Wang & Henderson, 2018). Moreover, their expectations are affected by information about the recipients’ social dominance and in-group membership. Toddlers expect a distributor to display ingroup favoritism when resources are scarce and an egalitarian sharing is not possible (Bian et al., 2018) and they look longer when a distributor favors a subordinate individual instead of a dominant one (Enright et al., 2017). This suggests that infants expected the distributor to favor dominant recipients, but it is also possible that they were focusing on what the dominant character might do after being denied a larger share of the resources.

Children and adults care not only about distributive justice, but also about *procedural justice*: they are concerned both about the final outcome of a distribution and about how a certain outcome was achieved (e.g., Grocke et al., 2015; Jacobs et al., 2022; Shaw & Olson, 2014; Stowe et al., 2022). At present, only one study investigated whether also toddlers are sensitive to violations of procedural fairness (Surian & Margoni, 2020). This study, however, did not present toddlers with distributive actions but with scenarios in which two puppets asked for help in opening a box to a fair or to an unfair helper. Following the requests for help, the fair helper provided help to both puppets after the same delay. By contrast, the unfair helper provided help immediately to one puppet, but left the other puppet waiting (asking three or five times) before receiving the requested help. Children looked longer at the procedurally unfair event, suggesting that they expected an equal solicitude and detected a violation of fairness.

Infants’ expectations of agents’ fairness are affected by observing prior violations in different moral domains. After seeing an agent performing a helping action, 10-month-olds expected her to perform a fair distribution, but when the agent first performed a hindering action, they no longer expected her to distribute fairly (Surian et al., 2018). These results suggest that infants are able to form representations of agents’ moral character (Sperber &  Baumard, 2012). Convergent results were found by Ting and Baillargeon (2021): at 25 months, fairness expectations are affected by observing both previous hindering actions, as in Surian et al., (2018), and actions that violate the in-group loyalty principle. Moreover, the link between the domain of harm and the domain of distributive unfairness appears to be bidirectional. Gill and Sommerville (2023) found that witnessing an agent’s distributive behaviors affected infants’ expectations of whether that agent would later help or hinder another agent. Overall, several findings from different labs indicate that the impartiality constraint is taken into account by infants and toddlers.

## Agency

The evaluation of fairness and justice should be constrained only to contexts and events in which agents modify the states of other agents. Some infant studies included control conditions in which recipients were replaced by inert objects or by inanimate puppets that do not move or talk. These conditions provide good controls as the inanimate puppets were perceptually identical to the self-moving puppets used in the experimental conditions. Infants in the first and second year saw an agent moving resources from one place to another place – like in the distributive events –, but those spatial changes did not modify the state of other agents (e.g., Geraci & Surian, 2011; Meristo et al., 2016; Sloane et al., 2012; Strid & Meristo, 2020). Consistently with the *agency* constraint, in these scenarios infants and toddlers did not show a preference for agents that performed symmetrical motions.

Infants individuate agents by attending to simple perceptual cues, such as autonomous motion (Decarli et al., 2020; Surian & Caldi, 2010). The evaluation of fairness requires the individuation of agents and such individuations and categorizations processes may exploit the representational resources provided by infants’ actional understanding (Leslie, 1994) and the agents core system of knowledge (Spelke, 2022). But studies on fairness evaluation suggest that infants represent agents as also endowed with sociomoral properties (Ting et al., 2020). Therefore, the representations of agents that infants generate during their evaluations appear to be richer that those provided by the core systems proposed by Spelke (2022).

The agency constraint requires that fairness evaluations should be linked to the individuation and understanding of agents. By studying how infants’ evaluations of distributions are intertwined with their understanding of agents one can gain further assessments of the agency constraint and shed some light on the mechanisms underpinning infants’ responses. One way to reason about agents is to attribute to them goals and preferences and take into account relevant physical constraints (Gergely & Csibra, 2003; Leslie, 1994; Woodward, 1998). Preverbal infants understand actions goal-directedness and react less negatively towards an adult that is unable to give them a toy than towards one that is teasingly unwilling to do so (Behne et al., 2005; Geraci et al., 2024; Luo, 2011; Sommerville et al., 2005). At 15 months they ascribe the goal of giving an object by taking into account cues about object possession (Tatone et al., 2019). Crucially, infants’ ability to attribute goals and preferences predicts, 5 years later, their intention-based moral judgments: children who, at 7 months, passed a well-known goal-attribution task (Woodward, 1998), performed better, at the age of 5 years, in a task involving intention-based moral judgments (Sodian et al., 2016).

Generating moral judgements by attending to agents’ intentions is a universal aspect of moral competence (Barrett & Saxe, 2021; Mikhail, 2022) that emerges very early in development (see Hamlin, 2013b, for findings in the help/harm domain). Preverbal infants attend not only to distribution outcomes, but also to distributors’ intentions. Strid and Meristo (2020) showed 10-month-olds simple scenarios involving one distributor and two recipients. After giving one strawberry to one of the recipients, the distributor failed to deliver the second strawberry either to the same or to the other recipient. In the test events, a third party approached one of the two distributors, and infants looked longer at the approach-unfair event, suggesting that they considered the distributors’ intentions and expected the third party to approach the distributor who tried to act fairly. Two further studies presented failed attempts to distribute windfall resources fairly and unfairly and found that young infants, aged 9 (Geraci et al., 2022) and 4 months (Geraci & Surian, 2023a) preferentially chose and looked at the fair distributor. This very early emergence satisfies the age requirement for core knowledge (Spelke, 2022).

Many studies found evidence showing that infants can go beyond the attribution of goals (or intentions) when reasoning about agents, they also take a mentalistic stance by attributing epistemic mental states (e.g., Choi & Luo, 2015; Onishi & Baillargeon, 2005; Surian et al., 2007). Mentalistic reasoning typically involves the representation of agents’ true and false beliefs (i.e., metarepresentations) to predict or make sense of their actions. Most experimental studies revealed metarepresentational skills in the second year (for reviews see Baillargeon et al., 2015; Scott & Baillargeon, 2017; Kampis et al., 2020; Sodian et al., 2020), but some studies reported positive evidence of mentalistic understanding also in preverbal infants (Choi et al., 2022; Kampis et al., 2015; Kovács et al., 2010; Southgate & Vernetti, 2014). Sloane et al. (2012) and Meristo and Surian (2013) found evidence suggesting that infants can reason in a mentalistic way when they process scenarios involving resources distributions. Sloane et al. (2012) presented a task designed to assess toddlers’ merit-based evaluations. Sloane et al. (2012) found that toddlers generally expect compensations to be commensurate with efforts. However, participants did not show evidence of having these expectations when the distributor ignored relevant information. In that study, toddlers first watched a distributor assigning a task to two individuals (e.g., tiding up the room). Then she performed an equal distribution towards the same individuals, while ignoring that only one of them had obeyed and completed the task. In Meristo and Surian (2013) study, ten-month-old infants were shown an agent rewarding a fair or an unfair distributor. When it was clear that the rewarding agent had watched the distributive actions performed by the distributor, infants were surprised to see that she rewarded the unfair distributor. By contrast, when it was clear that the rewarding agent could not have seen the distributive actions, infants had no specific expectation about which distributor would receive the reward. These findings suggest that infants and toddlers took into account distributors informational states to generate relevant behavioral expectations (for related results showing infants’ mentalistic reasoning in the evaluations of hinderers and helpers, see Choi & Luo, 2015; Hamlin, 2013b; Woo et al., 2017; Woo & Spelke, 2022).

In sum, several findings indicate that infants take into account the agency constraint when they evaluate distributive actions. Moreover, the links between infants’ early sociomoral evaluations and their ability to reason about agents’ intentions and epistemic mental states provide some support for models that emphasize developmental continuity rather than radical conceptual changes (Margoni & Surian, 2017).

## Obligatoriness

The feeling of obligation may have its roots in reciprocity mechanisms that allowed the evolution of cooperative hunting in our Pleistocene ancestors and provided the foundations for the ability to learn social norms quickly and spontaneously (Tomasello et al., 2012). Expectations of reciprocity and fairness emerge early in human infants (Jin et al., 2024) and rely on principles that are quite different from those that operate in other domains. While abstract principles in the core domains of objects, places and numbers lead to expectations about what is likely to occur in a given context, sociomoral principles mandating fairness and prohibiting harm generate prescriptions about what ought to occur and how to evaluate actions as good and right, or bad and wrong. The *obligatoriness* constraint is a crucial aspect of a core concept of justice, but it is a very difficult one to assess in infants and toddlers. For this reason, it is a source of long-lasting controversies. Many studies showed that infants prefer prosocial to antisocial individuals and expect equal or merit-based distributions, but we need to distinguish preferences and expectations from feelings of obligation. An evaluation of fairness and justice brings with it a sense of obligation that preferences or expectations do not necessarily include.

Infants selectively associate fair or unfair actions with expressions of praise or admonishment (DesChamps et al., 2016). Infants at 15 months watched a fair and an unfair distributor side by side and, while they were hearing expressions of praise, they looked longer at the unfair agent than at the fair one, showign a reliable mismatching effect. By contrast, when they were hearing admonishments they showed no reliable visual preference for for one of the two agents. At 13 months, infants showed a reliable preference for the unfair agent while they were listening to admonishments, showing a matching rather than a mismatching effect. Infants also link fair and unfair actions with the deliverance of rewards (Meristo & Surian, 2013) and punishment (Geraci & Surian, 2023b. In Meristo and Surian (2013) study, 10-month-olds were surprised to see that a third-party chose to reward an unfair distributor (by giving a strawberry). Likewise, Geraci and Surian (2023b) found that 9-month-olds infants, in test trials that showed simultaneously a fair and an unfair distributor, were surprised when a third-party chose to reward the unfair agent. Infants were also surprised when they watched the third-party chose to deliver a physical punishment to the fair rather than the unfair distributor by hitting him with a stick. These findings provide further evidence revealing the sociomoral nature of infants’ evaluations and suggesting an early-emerging understanding of retributive actions. Further evidence in line with this conclusion was reported by Ziv et al. (2021) who found a spontaneous tendency to deliver rewards to fair rather than to unfair agents by selectively touching the area of a screen that activated a recording of vocal expressions of praising. Consistently, Stavans and Baillargeon (2019) found that 17-month-olds expect a leader to intervene and rectify the situation when an ingroup member takes more than its share of a windfall resource, suggesting a sense that a wrong has been committed that needs to be corrected. Moreover, they hold no such expectation for a non-leader, who might be scared of reprisals from the wrongdoer.

These reactions suggest that infants and toddlers hold expectations about third parties’ retributive behaviors enacted towards fair and unfair distributors and they can even act to deliver such retributions. Is this showing that infants link fairness to obligatoriness? Only weakly, since punishments and rewards do not necessarily derive from moral disapprobation or approval, they can also be delivered, more simply, out of dislike or a wish for behavioral control.

For some authors, a feeling of obligation can only be revealed by explicit verbal judgments, justifications, and protests that display a normative vocabulary (Dahl, 2018; Paulus, 2020). Notice, however, that even if you subscribe to this view, you still need to clarify what young children mean when they use normative and evaluative terms in their utterances (Franchin et al., 2019), and explain how they succeed in acquiring the semantics of those abstract words. One possibility is that the early emergence of some core sociomoral concepts allows toddlers to fast map the meaning of their first normative and evaluative words. Further evidence on the early emergence of a sense of obligation may be obtained, in future studies, by recording how infants react to people performing actions that we see as obligatory or as supererogatory (McManus et al., 2020; Ting, 2020). Overall, the existing evidence is consistent with an early sensitivity to obligatoriness, but the proofs are not conclusive and need to be strengthened in future works.

## Conflicting Claims and Interpersonal Conflicts

Justice and fairness are usually invoked when conflicting claims and interpersonal conflicts arise and when people try answer questions like ‘How one ought to divide these resources given the existing conflicting claims?’. By contrast, when agents’ goals do not compete, but rather converge, efficiency considerations may take central stage (‘What is the best way to reach our shared goals?’). Also, David Hume claimed that in a hypothetical state of abundance where any conflict would turn out useless “the jealous virtue of justice would never once have been dreamed of” (Hume, 1975, p. 184; cit. in Miller, 2021; see also Rawls, 1971). So, real or hypothetical interpersonal conflicts about resource distributions, welfare and rights, are probably the typical contexts in which the concept of justice is invoked. Is there any evidence, in the infant literature, for a link between conflicts and the activation of the concept of justice? There is abundant evidence that infants attribute goals, intentions and other mental states to agents, as we discussed above, and there is also some evidence that they, by 10–13 months, can represent interpersonal conflicts (e.g., Mascaro & Csibra, 2012; Thomsen et al., 2011) but, at present, we have no proof that their ability to detect conflicts is linked to the evaluation of distributive fairness.

It is plausible that, when infants are faced with the simple scenarios used to assess their sense of fairness, they represent the recipients as having an interest in receiving the available resources and as having conflicting claims, goals or desires about the final outcome of the distribution. However, direct evidence for this has not been reported yet. It would be therefore of great value to study whether the presence of a salient conflict between agents and claims primes infants’ evaluations of fairness. Another way to investigate this question would be to present infants with scenarios in which it is clear that there are no conflicting claims and test whether this would affect their evaluations and expectations.

The fact that researchers have, so far, paid less attention to the conflicting claims component than to the other three components may not be without a reason. Conflicting claims do not appear to be, like the other three aspects, a necessary component of JUSTICE, but rather a contextual feature affecting its instantiation. While the need to solve social conflicts is likely to have played, in our ancient past, a crucial role in the evolution of a sense of fairness (Tomasello et al., 2012), we do not know whether the perception of conflicting claims, or an interpersonal conflict, facilitates the activation of the concept of justice. To test this possibility, future studies could build on what we know about infants’ understanding of social power and conflict (Margoni et al., 2018; Pun et al., 2016).

## A Comparison with Related Theoretical Proposals

Our proposal about infants’ sense of fairness and the core concept of justice dovetails with some previous proposals put forward by leading authors in this research field. Baillargeon, Bian, Buyukozer Dawkins, Ting, and their colleagues claimed that early moral cognition includes four moral principles (fairness, harm avoidance, in-group support, and authority) and four dedicated moral concepts (obligations, status, circle, and character; Baillargeon et al., 2015; Buyukozer Dawkins et al., 2019; Ting et al., 2020). These principles are similar to those posited in adult studies by the Moral Foundations Theory (Graham et al., 2013; Haidt, & Joseph, 2007) and they do not determine who is virtuous, but what actions are obligatory, forbidden or permissible, thus generating expectations about what an individual will (and should) do in certain situations. Infants possess a concept of moral obligation and rely on an “equity-based principle of fairness: all other things being equal, individuals are expected to give others their just deserts—that is to treat them as they deserve to be treated in the situation at hand” (Ting et al., 2020; p. 44). These principles apply to a variety of situations, including those that involve the division of windfall resources, the dispensation of rewards to individuals commensurate to their relative effort, and the delivery of punishments to disobedient agents. Also, “infants conceptualize the various principle-based expectations as *obligations* that specify how individuals should act toward others (e.g., individuals should act fairly)” (Ting et al., 2020; p.49, emphasis in the original). All this is coherent with the proposal we put forward in the present paper. So, what do we gain by positing also a core concept of justice? The principle of fairness, proposed by Baillargeon and her colleagues, prescribes that individuals should act fairly. Needless to say, here ‘should’ means ‘ought to’, so such a principle requires the concepts of INDIVIDUAL, OUGHT, and ACTING FAIRLY. Ting et al. (2020) discuss in depth the first two (INDIVIDUAL, as an agent endowed of moral status, and OBLIGATION), but not the concept of JUSTICE as a property of actions and agents. This concept is one of the necessary building blocks of the principle of fairness. Thus, our proposal is not meant to replace, but rather to integrate their broader model.

Overall, our proposal is also coherent with many works by Hamlin, Bloom, Tan, Woo and Wynn (Bloom, 2014; Hamlin, 2013a, b; Hamlin et al., 2007; Wynn & Bloom, 2014; Tan & Hamlin, 2022; Woo et al., 2022; Tan et al., 2018) where we read that “humans’ core system of knowledge include primitive abilities to understand and evaluate morally relevant actions (…). We term these abilities a ‘moral core’.” (Woo et al., 2022; p. 41). While their experimental work has been focused, primarily, on infants’ evaluation of actions that help or hinder other agents’ attempts to fulfill their goals, our work has been focused mainly on the domain of distributive fairness and it expands, in a consistent way, their proposals. However, in reviewing the available evidence on infants’ evaluation in different moral domains, Woo and Hamlin (2022) reached the conclusion that while there are many findings showing a preference for helping agents (over hindering ones) in the first year of life (e.g., Hamlin et al., 2010), a preference for fair agents has been found in the second, but not in the first year (Burns & Sommerville, 2014; Geraci & Surian, 2011; Lucca et al., 2018), and this suggest a different developmental onset for the principles prohibiting harm and prescribing fairness. Since recent works found expectations of fairness at 4 months (Buyukozer Dawkins et al., 2019) and a preference for fair individuals in infants aged 4 and 9 months (Geraci & Surian, 2023a, 2023b; Geraci et al., 2022), we believe that the available evidence suggests that both the harm-avoidance and the fairness principles emerge in the first year, at approximately the same time. Moreover, we suspect that these two domains may be more closely linked than it has been assumed by the Moral Foundations Theory (Graham et al., 2018). It is possible that the delivery of an unfair distribution is always perceived, implicitly and automatically, as involving harm, similarly to what happens when people read scenarios involving impure actions, even if such actions are described and judged explicitly as totally safe and harmless (e.g., Gray et al., 2014; Osler et al., 2024). In this light, it would also be useful to investigate, in future studies, the links between infants’ sense of fairness and the early emergence of concerns for others (Davidov et al., 2013).

Sommerville, Lucca, Burns, Enright, Ziv and their colleagues reported many seminal findings that are consistent with our view, but there are also some noteworthy differences between their theoretical perspective and ours. They reported infants’ expectation of fairness (Schmidt & Sommerville, 2011; Sommerville et al., 2013; Enright et al., 2017) as well as infants’ preference for fair agents (Burns & Sommerville, 2014; Lucca et al., 2018; see Sommerville, 2018, 2023, for reviews), but they also found some developmental changes that, in their view, support a constructivist perspective (Ziv & Sommerville, 2017). We instead think that those findings do not support selectively neither a constructivist nor a nativist view, as they are mute about the domain generality or specificity of the mechanisms underlying the early acquisition of sociomoral preferences. By contrast, the very early emergence of sociomoral evaluations, expectations and preferences do provide some evidence for the nativist views and they are at odds with constructivist theories, particularly with those that emphasize the role of peer interactions (Killen, 2018).

The domain specificity of the acquisition mechanism is a central issue in any attempt to adjudicate between nativist and constructivist models of cognitive and moral development (Laurence & Margolis, 2024). A sophisticated attempt to take seriously this issue can be found in the authors that endorse the linguistic analogy (Rawls, 1971) and posit the existence of a Universal Moral Grammar (Mikhail, 2007). Like the Universal Grammar, proposed to account for native language acquisition, the Universal Moral Grammar would provide the representational resources needed to parse morally relevant actions and contexts, allowing children to rapidly and spontaneously acquire the morality that characterizes the culture in which they are immersed. This view focused on representations needed for deontic reasoning and the judgment of intentional and accidental harming actions as prohibited or permissible, as a function of their outcomes and intentions (e.g., Dwyer et al., 2010; Levine et al., 2018). While our proposal is consistent with this account, more theoretical work and a great deal of methodological ingeniousness are necessary to show how this approach can be fruitfully extended to account for infants’ sense of fairness.

## Future Directions

Simple egalitarian expectations appear very early, in infants as young as 4 months, and merit-based evaluations emerge in toddlers. This very early emergence and fast development is more consistent with an initial state that is endowed with core knowledge and domain-specific developmental mechanisms than with views that deny any knowledge of abstract principles and posit only slow, domain-general associative learning. The evidence we reviewed here provides some initial support for an early emerging core concept of justice. It does not rule out, however, a role for constructivist developmental processes that may be responsible for important conceptual changes occurring later, during childhood and adolescence (Killen et al., 2022). Also, we cautiously say that this evidence should be seen as providing ‘initial support’ for a core concept of justice since it is neither unequivocal nor conclusive. More work is needed to consolidate these findings via multi-lab replications (e.g., Lucca et al., 2021), to deepen our understanding of the information processing mechanisms underpinning infants’ evaluations and expectations (Stahl & Feigenson, 2019), and to further assess the role of environmental input and statistical learning (Ruffman et al., 2012). Longitudinal studies could fruitfully clarify the present findings by testing whether infants’ reactions to unfair distributions reliably predict the judgments that children will express verbally some years later (see Tan et al., 2018 for related results).

Relevant cross-cultural infant studies are, unfortunately, still rare. Only one published study assessed toddlers’ sense of fairness in non-WEIRD populations from small-scale societies and it reported remarkable cross-cultural differences. Meristo and Zeidler (2022) compared a group of 12 to 20-month-olds from Sweden with infants from Samburu and Kikuyo populations, two Kenyan ethnic groups. Swedish infants looked longer at unequal distributions, whereas Kikuyo infants looked equally at the two types of distributive outcomes and Samburu infants looked longer at equal distributions, suggesting an early effect of culture-specific experience. Further cross-cultural infant studies could be inspired by previous research on adults and children. Concerns for fairness and egalitarian principles in distributions appear to be a universal aspect of human moral competence (Boehm, 2012; Curry et al., 2019). However, a rich literature on adults’ economic reasoning and decision making has also revealed noteworthy cross-cultural variations on how people judge distributions and how they decide to share windfall resources (e.g., Henrich et al., 2005). Participants from WEIRD and from many small-scale societies may opt for an egalitarian rule when they participate in a sharing task, or when they are asked to judge the fairness of a distribution, but, as a second choice, children from the US favor merit-based rules while children from other cultures may favor need-based rules (e.g., Carson & Banuazizi, 2008; see also Huppert et al, 2019). A universal, early emerging principle of justice may mandate that one should treat like cases alike and different cases differently. If this principle is so formulated, it lacks determinant content, but it helps to order our experience, allowing the construction of a “theory of justice of which features of similarities and differences actually matter” (Shweder, 2004, p. 91).

Cross-cultural differences in behavioral choices are predicted by social factors such as the level of market integration (i.e., how often an individual is involved in buying or selling goods; Henrich et al., 2005). Young children from a great variety of cultures tend to behave more selfishly than adults in their sharing decisions, by keeping most of the resources for themselves (Cowell et al., 2017; Rochat et al., 2009), but by the age of 5 years they possess a sense of ownership that leads them to spontaneously respect others’ properties (Kanngiesser et al., 2019) and by mid-childhood they follow egalitarian rules and spontaneously choose to punish agents that behave selfishly in resource distributions (House et al., 2013, 2020). While we know that infants’ expectations of fairness are linked to their sharing decisions (Schmidt & Sommerville, 2011), and early sharing experiences predict infants’ later reactions to fair and unfair distributions (Ziv & Sommerville, 2017), there is much to study, both longitudinally and cross-culturally, to better describe and understand these links. No previous study has assessed whether infants can follow need-based principles to evaluate resource distributions and whether they can reason about procedural fairness in distributive contexts (Shaw & Olson, 2014).

A central and early emerging aspect of moral competence is the ability to tell moral norms from conventional norms, assuming that only moral norms are authority and context dependent (Turiel, 1983). Beliefs in the context and authority independence of moral norms are found in WEIRD as well as in small scale societies (Piazza & Sousa, 2016), although the expression of these beliefs by adults’ is affected by pragmatic factors (Margoni & Surian, 2021). Even toddlers react differently to moral and conventional transgressions (Smetana, 1984). Do they also see distributive and procedural unfairness as violations of a moral norm? The evidence reviewed here appear to support a positive answer, but recent results on preschoolers suggest they do not (Yucel et al., 2022) and therefore more direct tests are necessary.

We still know very little about how infants face situations that may involve conflicts between different principles, for example a conflict between fairness and respect for authorities or group loyalty (Bian et al., 2018; Ting et al., 2020; Woo & Hamlin, 2023), or a conflict between merit-based and need-based distributive rules. The evidence we reviewed above shows that infants and toddlers evaluate the fairness of many types of agents, including non-human agents. We need further investigations of how fairness and justice concerns are constrained by information about ingroup/outgroup membership, moral circle membership and agents’ hierarchical status.

## Conclusions

Humans’ success in adapting to the most diverse environments relies on the capacity to coordinate their actions with those of many other unrelated individuals and engage in sophisticated cooperative activities. We are ‘super-cooperators’ because, among other things, we can rely on abstract concepts that allow us to reason about agents and evaluate their actions, including their fairness (Baumard et al., 2013). It is plausible that the acquisition of these universal skills is underpinned by some proximate mechanisms and some universal norms that “evolved because they constitute the most effective means of upholding mutually beneficiary cooperative relations” (Krebs, 2008, p. 168). By emphasizing the role of these capacities in allowing complex cooperation, these views help to identify moral universals (Blake et al., 2015; Curry et al., 2019) and to explain the developmental time course of fairness concerns and sharing preferences (Sheskin et al., 2014).

Consistently with this evolutionary view, a growing body of evidence reveals the early emergence of a sense of fairness. Infants’ and toddlers’ expectations and preferences suggest that their evaluations of fairness manifest most of the signature features that are also found in children’s and adults’ moral evaluations. These evaluations are restricted to agents, they concern violations of both distributive and procedural norms, they rely on agents’ intentions and other mental states, they are linked to expectations of third-party punishment and rewards, and they even motivate rewarding actions. Moreover, the involvement of theory of mind undermines the viability of alternative accounts that tried to explain looking times results as solely due to the effect of low-level perceptual factors.

In the past decade, many studies have consistently provided support for the claim that infants’ and toddlers’ sociomoral competence is much richer than previously assumed. Here we proposed that it may even include a rudimentary sense of fairness that relies on a tacit concept of justice. These proposals posit the ‘developmental roots’ or the ‘seeds’ of fairness, but they are not meant to revive preformistic views, deny that developmental changes occur in childhood and adolescence, or suggest that the development of sociomoral skills is merely the unfolding of a genetic blueprint. By clarifying and extending these results in longitudinal and cross-cultural studies, future research will have the chance to broaden our understanding of how humans acquire a mature moral competence and develop their full-fledged ability to reflect on persons’ duties and rights.

## Data Availability

We do not analyze or generate any datasets, because our work proceeds as a review of the current literature, but does not provide a meta-analysis of the results reported in previous articles. One can obtain the details on these results and, in some cases, the access to the original data sets, from the references below.
